# Description of *Stieleria mannarensis* sp. nov., isolated from a marine sponge, and proposal to include members of the genus *Roseiconus* in the genus *Stieleria*

**DOI:** 10.1007/s10482-025-02106-8

**Published:** 2025-07-11

**Authors:** Nicolai Kallscheuer, Gaurav Kumar, Shabbir Ahamad, Sandhya Duddeda, Chintalapati Sasikala, Christian Jogler, Chintalapati Venkata Ramana

**Affiliations:** 1 Department of Microbial Interactions, Institute of Microbiology, Friedrich Schiller University, 07743 Jena, Germany; 2 Department of Plant Sciences, School of Life Sciences, University of Hyderabad, P.O. Central University, Hyderabad, 500046 India; 3 Cluster of Excellence Balance of the Microverse, Friedrich Schiller University, Jena, Germany; 4 Bacterial Discovery Laboratory, Centre for Environment, Institute of Science and Technology, JNT University Hyderabad, Kukatpally, Hyderabad, 500085 India

**Keywords:** *Planctomycetota*, Marine bacteria, *Pirellulaceae*, Sponge, Gulf of Mannar, Stieleriacines, Pangenomics

## Abstract

A pink-coloured, salt- and alkali-tolerant strain, JC639^T^, was isolated from a specimen of the marine sponge *Spheciospongia* sp. The novel isolate is motile, aerobic and forms oval to pear-shaped cells. It shares the highest 16S rRNA gene sequence identity with *Stieleria neptunia* Enr13^T^ and *Stieleria maiorica* Mal15^T^ in the family *Pirellulaceae* (phylum *Planctomycetota*). Optimum pH and temperature for growth are 8.0 (range 7.0–10.0) and 25 °C (range 10–30 °C), respectively. Major fatty acids are C_15:1_ω8c, C_16:0,_ C_18:1_ω9c, C_18:0_, and C_16:1_ω7c/C_16:1_ω6c. Major polar lipids are phosphatidylcholine and phosphatidylethanolamine. The genome of strain JC639^T^ has a size of 9.56 Mb and a DNA G + C content of 59.5%. The polyphasic analysis of the isolate supports its delineation from the known species in the genus *Stieleria*. We therefore introduce *Stieleria mannarensis* sp. nov., represented by JC639^T^ (= KCTC 72168^T^ = NBRC 113878^T^) as the type strain. Based on an analysis of established phylogenomic markers performed with all current members of the overlapping genera *Stieleria* and *Roseiconus*, we further propose to include the two so far characterized *Roseiconus* species in the genus *Stieleria*.

**Supplementary Information:**

The online version contains supplementary material available at 10.1007/s10482-025-02106-8.

## Introduction

Bacteria belonging to the phylum *Planctomycetota* have garnered significant attention in recent years due to their unique cell biology and ecological and evolutionary importance (Fuerst and Sagulenko 2011; Jogler et al. 2012; Shiratori et al. 2019; Wiegand et al. 2018; Wurzbacher et al. 2024). The phyla *Planctomycetota*, *Verrucomicrobiota, Lentisphaerota, Kiritimatiellaeota,* “*Candidatus* Omnitrophota” and *Chlamydiota*, form the PVC superphylum (Wagner and Horn 2006). They are widely distributed, and most described strains have been isolated from marine habitats. Members of the phylum are highly abundant on the surface of marine phototrophs like macroalgae (Kumar et al. 2024; Lage and Bondoso 2014) or seagrasses (Kohn et al. 2020a), sponges (Izumi et al. 2013; Kohn et al. 2020b; Kumar et al. 2021b), marine snow (DeLong et al. 1993), and cyanobacterial aggregates (Cai et al. 2013; Kallscheuer et al. 2021), where they can dominate bacterial biofilms. They are characterized by distinctive features not commonly detected in other prokaryotes, such as an enlarged periplasmic space, outer membrane complexes in the form of crateriform structures, and a non-FtsZ-based division mode (Boedeker et al. 2017; Rivas-Marin et al. 2020). Their genomes harbour giant genes, which might be responsible for the biosynthesis of small bioactive molecule or encode parts of structural components (Calisto et al. 2025; Graça et al. 2016; Wiegand et al. 2020). Members of the phylum *Planctomycetota* also have biotechnological relevance (Kallscheuer and Jogler 2021; Vitorino et al. 2024).

Sponges (phylum *Porifera*) are the second largest sessile component of coral reefs and evolved around 600 million years ago. Holobionts are formed when they build tight associations with a diverse range of microorganisms, such as bacteria, fungi, protists, and archaea (He et al. 2014; Hentschel et al. 2012; Moitinho-Silva et al. 2017; Thomas et al. 2016). Sponges serve as hosts for various microorganisms that can constitute for up to 60% of the total sponge biomass (Vacelet and Donadey 1977). Cultivation-independent studies, like metagenomic analyses and fluorescence in situ hybridization, revealed that planctomycetes are commonly associated with sponges and constitute 1–14% of the total microbiome associated with sponges (Kohn et al. 2020b; Najafi et al. 2018). Members of the phylum *Planctomycetota* are frequently isolated from various species of sponges (Kallscheuer et al. 2020c; Kohn et al. 2020b; Kumar et al. 2021b, c). Sponges of the genus *Spheciospongia* are abundantly found in the southern hemisphere. Symbionts associated with *Spheciospongia*, including *Planctomycetota*, play vital roles in the nitrogen cycle of coral reef ecosystems, e.g., during nitrification, denitrification, and nitrogen fixation (Delmont et al. 2018; He et al. 2021).

In the course of our investigation of the planctomycetal diversity across the Indian subcontinent, we described several novel genera and species from various geographical locations (Kumar et al. 2020b; Kumar et al. 2024; Lhingjakim et al. 2022; Sreya et al. 2023). Here, we describe another novel species isolated from a sponge sample found on the shore of Rameswaram. Rameswaram is located at the tip of the Indian peninsula and is situated on Pamban Island. The island is part of the Gulf of Mannar, a species-rich biosphere reserve on the eastern coast of India. Several cultivation- and metagenomics-based studies yielded data on the vast bacterial diversity in the Gulf of Mannar with *Planctomycetota* being one of the dominant bacterial phyla (9–10% of the bacterial community) (Muddukrishnaiah et al. 2022).

Since the here characterized isolate, strain JC639^T^, belongs to a novel species of the genus *Stieleria*, we used the occasion of this species description to include a more detailed phylogenetic analysis of all described members of the genus including strains belonging to the overlapping genus *Roseiconus* that was described shortly after the genus *Stieleria* (Kallscheuer et al. 2020b; Kumar et al. 2021a).

The current genus *Stieleria* is constituted by five validly described species, *S. maiorica* Mal15^T^*, S. neptunia* Enr13^T^*, S. sedimenti* ICT_E10.1^T^*, S. tagensis* TO1_6^T^*,* and *S. varia* Pla52n^T^ (Godinho et al. 2023; Kallscheuer et al. 2020b; Sandargo et al. 2020; Surup et al. 2020; Vitorino et al. 2022). The genus *Roseiconus* contains two species, *R. lacunae* JC635^T^*,* and *R. nitratireducens* JC645^T^ (Kumar et al. 2021a). For comparative taxonomic studies of the novel isolate JC639^T^ in the laboratory, the current closest relatives of JC639^T^, *S. maiorica* Mal15^T^ (= DSM 100215^T^)*,* and *S. neptunia* Enr13^T^ (= DSM 100295^T^) were included.

## Materials and methods

### Habitat and isolation

A sponge specimen belonging to the genus *Spheciospongia* was collected from the seashore of Rameswaram, India (GPS position: 9°16′10.0′′N 79°07′12.0′′E) in a 15 mL tube. The specimen was subjected to enrichment and cultivation of associated bacteria in modified M30 medium prepared as previously described (Kumar et al. 2020a). In the laboratory, the sampled sponge specimen was washed with sterile artificial seawater (ASW) and then homogenized with the help of sterile mortar and pestle. 100 mg of the homogenized tissue was suspended in 10 mL of sterile ASW and 200 µL of this solution was mixed with 10 mL medium in different 50 mL serum vials that were sealed with butylated rubber stoppers. The serum vials were then incubated for fifteen days at 25 °C to enrich members of the phylum *Planctomycetota* by exploiting their natural resistance to the supplemented antibiotics streptomycin (final concentration 400 mg/L), and ampicillin (final conc. 200 mg/L). 25 mg/L cycloheximide was supplemented to prevent fungal growth. After the incubation period, 100 µL of the enriched sample was plated on solidified modified M30 medium and was further incubated at 25 °C. After two weeks of incubation, pink colonies appeared along with white colonies (that had already appeared within 2 days). The pink colonies were purified through repeated streaking. A pure culture of the strain designated JC639^T^ was maintained on agar plates by repeated sub-culturing and preserved at 4 °C. The axenic culture was grown in the above-mentioned medium without antibiotics, unless otherwise stated.

### Genomic DNA isolation and sequencing

Genomic DNA was isolated from the axenic bacterial culture using the Nucleo-pore gDNA Fungal Bacterial Mini Kit (M/s. Genetix Biotech Asia Pvt. Ltd, India) and was used for PCR-based amplification of the 16S rRNA gene and genome sequencing. The PCR was performed using primers F40 (Köhler et al. 2008) and R1388 (Stackebrandt et al. 1993). Purified PCR products were sent to AgriGenome Pvt. Ltd. (Kochi, India) for 16S rRNA gene sequencing. The obtained sequence was used for a BLAST search analysis in the EzBioCloud (Yoon et al. 2017). Whole-genome sequencing (WGS) was outsourced to Agri Genome Pvt. Ltd, Kochi, India. WGS was carried out on an Illumina HiseqX10 platform and paired-end libraries were generated with a sequence coverage of 100x. De novo assembly was performed using Unicycler (Wick et al. 2017) with default k-mer sizes. The assembled genome was checked for any possible contamination using the ContEst service of EzBiocloud (Yoon et al. 2017) and annotated using RAST (Rapid Annotation using Subsystem Technology) (http://rast.theseed.org/FIG/rast.cgi).

### Phylogenetic inference and genome-based analyses

The 16S rRNA gene sequence extracted from the genome was used for the identification of the current closest relatives using BLASTn. A maximum likelihood 16S rRNA gene sequence-based phylogenetic tree was computed for the novel strain, the described type strains of all species in the current phylum *Planctomycetota* (as of January 2025) and 16S rRNA gene sequences extracted from available genomes from yet undescribed members of the genera *Stieleria* and *Roseiconus* based on NCBI’s Genome Browser. The alignment of the 16S rRNA gene sequences was performed with ClustalW (Thompson et al. 2003) and Fast-Tree was used for tree reconstruction with 1000 bootstrap replications (Price et al. 2009). Three sequences from strains outside of the phylum *Planctomycetota*, but part of the PVC superphylum, namely *Opitutus terrae* (NCBI acc. no. AJ229235), *Kiritimatiella glycovorans* (acc. no. NR_146840) and *Lentisphaera araneosa* (accession number NR_027571), served as outgroup. The multi-locus sequence analysis (MLSA)-based phylogeny was performed using autoMLST with 500 bootstrap replicates (Alanjary et al. 2019). The analysis included all genomes of strains belonging to the current genera *Stieleria and Roseiconus* and the genomes of *Rhodopirellula baltica* SH1^T^ (GenBank acc. no. BX119912.1), *Pirellula staleyi* DSM 6068^T^ (acc. no. CP001848.1) and *Blastopirellula marina* DSM 3645^T^ (acc. no. GCA_000153105.1) (all belonging to the family *Pirellulaceae*) served as outgroup. Phylogenetic trees were visualized with iTOL v6 (Letunic and Bork 2021). The 16S rRNA gene sequence similarity matrix was obtained with TaxonDC (Tarlachkov and Starodumova 2017) based on the ClustalW alignment that was also used for the construction of the phylogenetic tree. Average amino acid identities (AAI) and average nucleotide identities (ANI) were calculated using respective scripts of the enveomics collection (Rodriguez-R and Konstantinidis 2016). Additional phylogenetic markers, i.e. *rpoB* sequence similarity, percentage of conserved proteins (POCP) and digitally-derived genome-to-genome distances (dDDH) were calculated as described (Bondoso et al. 2013; Meier-Kolthoff et al. 2021; Qin et al. 2014). The pangenome of the selected strains was calculated with anvi’o v.8 (Delmont and Eren 2018). Biosynthetic gene clusters were analysed with antiSMASH v. 7.1 (Blin et al. 2023), exported and visualized with clinker (Gilchrist and Chooi 2021).

### Physiological analyses

For determining organic carbon substrate utilization, *N*-acetylglucosamine (NAG) was replaced with (NH_4_)_2_SO_4_ (0.1%, w/v) as the nitrogen source in modified M30 medium and different organic carbon substrates were used at a concentration of 0.1% (w/v). For nitrogen source utilization, *N*-acetylglucosamine (NAG) was replaced with 0.1% (w/v) glucose as carbon source and growth was tested with different nitrogen substrates at a concentration of 0.1% (w/v). Utilization of both, organic carbon and nitrogen substrates, was tested in glass test tubes (25 × 250 mm) containing 10 mL of liquid medium. NaCl tolerance (final NaCl concentrations of 1–10%, w/v) was tested at 25 °C and pH 8.0. The optimal temperature (5, 10, 15, 20, 25, 30, 35, 40, 45, 50 °C) for growth was tested in M30 medium at pH 8.0. The pH range (4.0, 5.0, 6.0, 7.0, 8.0, 9.0, 10.0) for growth was tested at 25 °C in buffered modified M30 medium (Bondoso et al. 2015). The enzymatic activity pattern was assayed using the API ZYM kit (Biomerieux, France) following the manufacturer’s protocol.

### Chemotaxonomic characterization

For fatty acid analysis, exponentially-growing cells were harvested by centrifugation (10,000 g, 15 min, 4 °C) at a cell density of 70% of the maximum optical density (100% = OD_660_ of 0.9). Cellular fatty acids were methylated, separated and identified according to instructions for the Microbial Identification System [Microbial ID; MIDI 6.0 version; method, RTSBA6) (Sasser 1990) which was carried out by Royal Research Labs, Secunderabad, India. Polar lipids were extracted, separated and characterized as previously described (Kates 1972; Oren et al. 1996). Polyamines were extracted and identified according to a published method (Kumar et al. 2020a).

### Microscopy

Cell morphology, like size and shape, and cell division were observed using scanning electron microscopy (SEM) (Olympus BH-2/Carl Zeiss LSM880/Philips XL3O). Transmission electron microscopy (TEM) (Zeol JEM2100, Japan) was performed to analyse cross sections of the cells.

### 16S rRNA gene sequence and genome accession numbers

The 1532 bp 16S rRNA gene sequence of strain JC639^T^ was deposited under GenBank accession number LR132063. The whole genome sequence of strain JC639^T^ is deposited at the NCBI database under accession number JACEHH010000000.

## Results and discussion

### Phylogenetic inference of the novel isolate

**Fig. 1:**
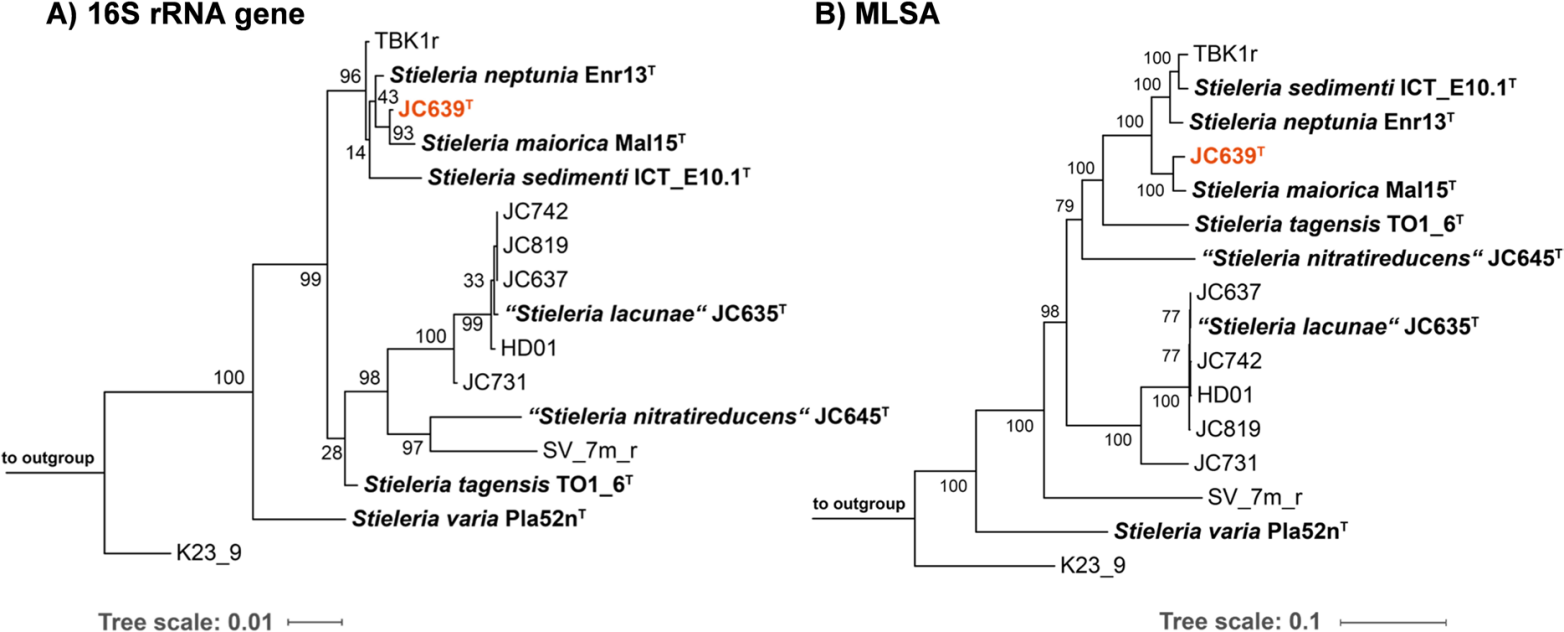
Maximum likelihood phylogenetic trees. **A** Maximum likelihood phylogenetic tree based on 16S rRNA gene sequences showing the phylogenetic relationship of strain JC639^T^ and other closely related members of the genus *Stieleria*. FastTree was used for tree reconstruction with 1000 bootstrap replications. Bar, 0.01 substitutions per nucleotide position. **B** Multi-locus sequence analysis (MLSA)-based phylogenetic tree. The phylogenetic tree based on MLSA shows the position of the described strain JC639^T^ in relation to its closest described neighbours of the genus *Stieleria.* The tree was computed based on a set of at least 30 single-copy gene-encoded proteins in a maximum likelihood approach with 500 bootstrap replications using the tool autoMLST (see Material and methods section for details). Bootstrap values are given at the nodes (in %). Bar, 0.1 substitutions per amino acid position. The used outgroups are described in the Material and methods sections. Both phylogenetic trees were visualized with iTOL v6. The type strains of the former *Roseiconus* species *R. lacunae* and *R. nitratireducens* are shown as *Stieleria* species (comb. nov.) in inverted commas

**Fig. 2:**
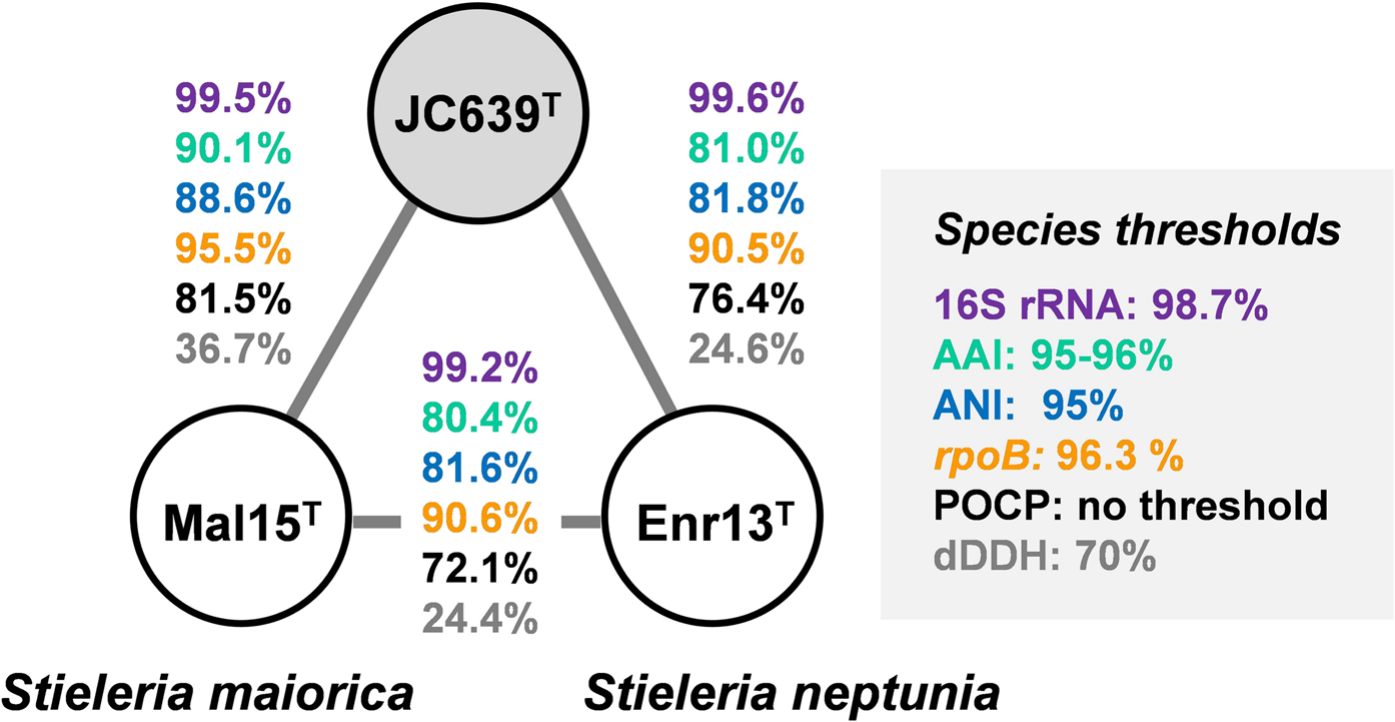
Comparison of phylogenetic markers for species delineation of the isolate JC639^T^. Markers used: 16S rRNA gene sequence identity (16S), average nucleotide identity (gANI), *rpoB,* average amino acid identity (AAI), percentage of conserved proteins (POCP) and digital DNA-DNA hybridization (dDDH)

The phylogenetic position of the novel isolate JC639^T^ was assessed based on 16S rRNA gene sequence- and MLSA-based maximum likelihood phylogenetic trees (Fig. 1). A delineation of the strain from known taxa was performed using five phylogenetic markers (16S rRNA gene sequence similarity, AAI, ANI, *rpoB* similarity, POCP and dDDH), taking the established threshold values for the phylum *Planctomycetota* into consideration (Fig. 2). In both trees, strain JC639^T^ clusters with members of the genus *Stieleria*, close to the type strain Mal15^T^ of the type species *S. maiorica* (Kallscheuer et al. 2020b) (Fig. 1A, B). The 16S rRNA gene sequences of JC639^T^ and Mal15^T^ differ in only seven nucleotide positions. This yields a sequence similarity value of 99.5% that falls above the proposed threshold of 98.7% (Yarza et al. 2014). However, 16S rRNA gene sequence similarities should be treated with caution for members of the phylum *Planctomycetota* since strains with near-identical 16S rRNA gene sequences have been assigned to different species based on additional phylogenetic markers in the past (Kohn et al. 2020b). Indeed, also in the case of strain JC639^T^ the 16S rRNA gene sequence similarity alone did not have sufficient resolution for species delineation. While consistently identifying *S. maiorica* Mal15^T^ as the current closest relative of strain JC639^T^, the other markers yielded values below the accepted thresholds (Kim et al. 2014; Luo et al. 2014; Qin et al. 2014) (Fig. 2). The POCP value is listed additionally although it is typically only applied for the delineation of genera. Hence, the overall analysis supports the assignment of strain JC639^T^ to a novel species in case that this is further supported by morphological, physiological and chemotaxonomic differences.

### Morphological and physiological analyses

**Fig. 3:**
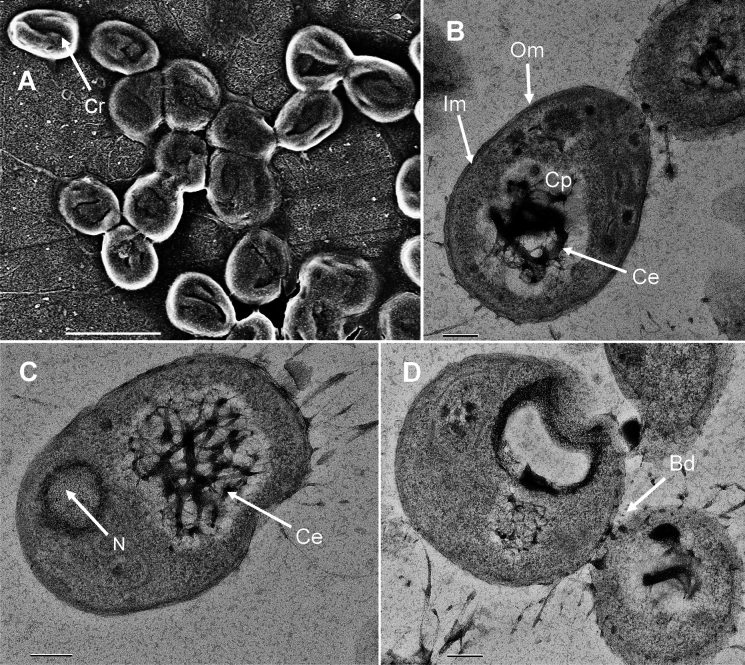
Electron microscopy images. **A** SEM image showing aggregation of the cells and presence of crateriform structures (Cr). The scale bar represents 2 µm. **B**–**D** TEM images depicting the late division stage of cells (budding, Bd) and the planctomycetotal cell plan with outer membrane (Om), inner membrane (Im), cytoplasm (Cp), condensed nucleoid (N) and potential cytoskeletal elements (Ce). The scale bar represents 0.1 µm

SEM images show aggregates and planktonic cells of strain JC639^T^ (Fig. 3). Cells are oval to pear-shaped (length: 1.5 ± 0.2 × width: 1.1 ± 0.2 μm) with crateriform structures distributed all over the surface (Fig. 3A). TEM images show the late stage of dividing cells by asymmetric division (budding), in which the mother and the emerging daughter cell are still connected (Bd, Fig. 3D). Additional images (Fig. 3B) point towards a Gram-negative cell plan with outer membrane (Om), inner membrane (Im) and a condensed nucleoid (N), the latter being typical for members of the phylum (Fig. 3B, C). In the cytoplasm, potential cytoskeletal elements (Ce) were observed (Fig. 3B, C). Such structures of yet unknown function seem to be randomly distributed throughout the cytoplasm cavities. The presence of related elements has been reported in some planctomycetal species before (Lage et al. 2013; Schubert et al. 2020).

Collected data on the growth behaviour with different carbon or nitrogen substrates as well as pH range, temperature and NaCl tolerance is summarized in the species description protologue and/or Table 1. The results of the analysis of carbon and nitrogen source utilization of *S. maiorica* Mal15^T^ and *S. neptunia* Enr13^T^ used for comparison are provided in the Supporting Information.

**Table 1:** Differential characteristics among strains JC639^T^, *S. maiorica* Mal15^T^, and *S. neptunia* Enr13^T^

Characteristics	JC639^T^	Mal15^T^	Enr13^T^
*Phenotypic features*			
Cell size (length x width, µm)	1.5 ± 0.2 × 1.1 ± 0.2	1.9 ± 0.2 × 1.4 ± 0.2	1.6 ± 0.1 × 1.1 ± 0.1
Cell shape	Pear-shaped to ovoid	Round to pear-shaped	Round grain rice-shaped to round
Pigmentation	Pale pink	Pink to salmon	Pink to salmon
Temperature range	10–30 (25)	11–37 (35)	9–35 (28)
(optimum) (°C)			
pH Range (optimum)	7.0–10.0 (8.0)	5.5–9.0 (7.5)	6.5–9.0 (7.5)
*Enzyme activities*			
Alkaline phosphatase	+	+	−
Esterase Lipase (C 8)	+	+	−
Valine arylamidase,	+	+	−
β-galactosidase	+	−	−
β-glucosidase	−	+	−
α-mannosidase	−	+	+
*Carbon source utilization*			
Lactose	+	+	−
sucrose	+	−	+
Na-pyruvate	+	−	−
starch	−	+	+
Sorbitol	−	+	+
*Nitrogen sources utilization*			
L-leucine	+	−	−
L-arginine	+	−	+
L-serine	+	+	−
L-isoleucine	−	−	+
DL-threonine	−	+	+
*Major fatty acids (*> *5%)*			
C_15:1_ω8c	+	−	−
*Polyamines*			
Homospermidine	+	−	+
Putrescine	+	−	−
Spermidine	−	+	−
*Genomic features*			
Genome size (bp)	9,558,029	9,894,293	10,975,817
DNA G + C (%)	59.5	59.3	58.9
Genes	6,636	6,727	7,502
Genes/Mbp	694	680	684
Protein-coding genes	6,576	6,696	7,441
Protein-coding genes/Mbp	688	677	678
Hypothetical proteins	1,577	2,900	3,144
Hypothetical proteins (%)	24.0	43.3	42.3
Coding density (%)	87.4	86.9	86.0
CRISPR arrays	2	1	2
rRNA genes (5S-16S-23S)	1-1-1	2-3-2	2-3-2
tRNA genes	67	73	88

Data taken from this study. + , positive., − , negative

### Chemotaxonomic characterization

For all three strains, JC639^T^, Mal15^T^ and Enr13^T^, major proportions of the fatty acids (> 5%) are C_16:0,_ C_18:1_ω9c, C_18:0_, and C_16:1_ω7c/C_16:1_ω6c (Table S1). C_15:1_ω8c (10.2%) is exclusively present in strain JC639^T^ (Table S1). Polar lipids of strain JC639^T^ are phosphatidylcholine (PC), phosphatidylethanolamine (PE), two unidentified phospholipids (PL1,2) and two unidentified lipids (L1,2) (Fig. S1). Polar lipid analyses for the type strains of *S. maiorica* (Mal15^T^) and *S. neptunia* (Enr13^T^) were not performed. The polyamine homospermidine is present in strain JC639^T^ and the type strain of *S. neptunia*. Putrescine and spermidine are exclusive for strain JC639^T^ and *S. maiorica* Mal15^T^, respectively (Fig. S2).

### The analysis of phylogenetic markers suggests that described *Stieleria* and *Roseiconus* species belong to a single genus

**Fig. 4:**
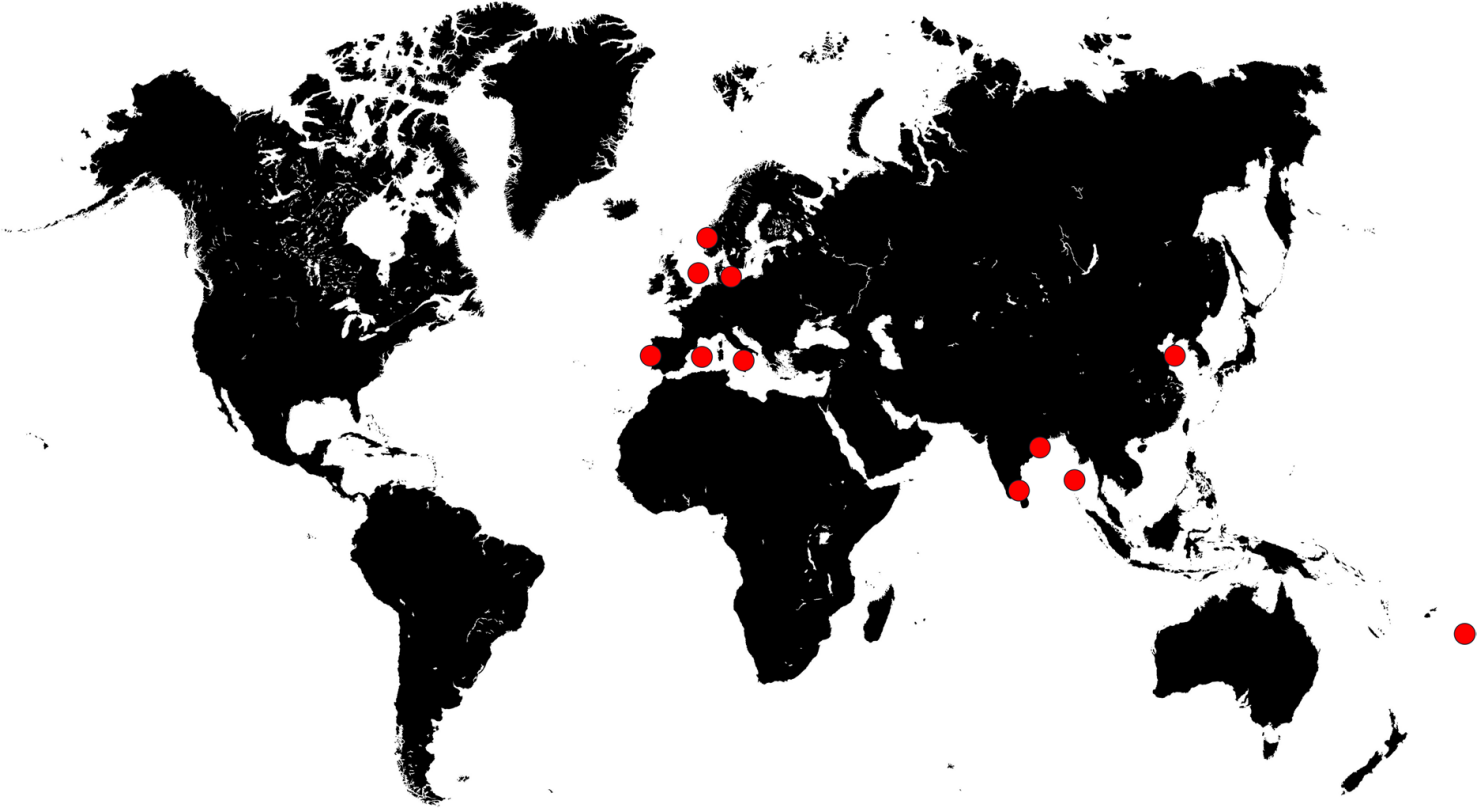
Sampling locations of *Stieleria* and *Roseiconus* strains. The world map illustrates the sampling spots, from which the investigated strains belonging to the genera *Stieleria*/*Roseiconus* have been isolated (see Table S2 for details)

In the framework of this species description, a phylogenetic comparison of all current members of *Stieleria* and *Roseiconus* was included. Members of both genera have been isolated from various locations worldwide (Fig. 4, Table S2) and frequently clustered together in recently published phylogenetic trees (Godinho et al. 2023; Øvreås et al. 2024). The here performed analyses were conducted based on the RefSeq-annotated genomes available from NCBI. The current genus *Stieleria* harbours five validly described species. In the chronological order of effective publication, the species and type strains are *S. maiorica* Mal15^T^ (Kallscheuer et al. 2020b), *S. neptunia* Enr13^T^ (Sandargo et al. 2020), *S. varia* Pla52n^T^ (Surup et al. 2020), *S. sedimenti* ICT_E10.1^T^ (Vitorino et al. 2022) and *S. tagensis* TO1_6^T^ (Godinho et al. 2023). The current genus *Roseiconus* is constituted by two validly published species, *R. nitratireducens* (type strain JC645^T^) and *R. lacunae* (type strain JC635^T^) (Kumar et al. 2021a). The *Stieleria* genus protologue has been published only shortly earlier than the *Roseiconus* protologue and in the context of the discovery of small molecules named stieleriacines (Kallscheuer et al. 2020b). Additional, not effectively published strains with an available genome sequence at NCBI falling into either of the two genera include strains TBK1r, SV_7m_r and K23_9 with provisional species names (Øvreås et al. 2024; Storesund et al. 2018; Wiegand et al. 2020), *Stieleria* sp. HD01 (Gao et al. 2024) and the non-published strains JC637, JC731, JC742 and JC819. Strain K23_9 did not re-grow after repeated inoculations and is no longer available as axenic culture (only the genome sequence is available for analyses). Taken together, the 16 mentioned strains (including JC639^T^) were included in the phylogenetic analyses. The same markers as for the delineation of strain JC639^T^ were used. A POCP value of > 50% is typically obtained for species belonging to the same genus. This value is in line with the results of the all-vs-all comparison of POCP values for the 16 genomes, in which only a single value of the 240 single comparisons (value of 49.2%) fell slightly below the 50% genus threshold (Table S3). Similarity values of a 1300 bp partial sequence of the gene *rpoB* encoding the β-subunit of RNA polymerase are used for phylogenetic inference in the class *Planctomycetia* with genus and species threshold (range) values of 75.5–78.0% and 96.3%, respectively (Bondoso et al. 2013; Kallscheuer et al. 2020d). No single value fell below the lower genus threshold value of 75.5%, supporting a relationship on genus level for all 16 strains (Table S4). Values obtained during the analysis of 16S rRNA gene sequence similarities and AAI were higher and only in exceptional cases slightly lower than the genus threshold values of 94.5% and 60%, respectively (Table S5 and S6). Thus, the four phylogenetic markers with available genus threshold values confirm the relationship of the 16 strains on the level of a single genus. Based on these findings, the current *Roseiconus* species should be included in the genus *Stieleria* and therefore reclassified as *Stieleria lacunae* comb. nov. and *Stieleria nitratireducens* comb. nov.

To check for the correct assignment of the not yet effectively published strains to the so far described species, the respective species thresholds were taken into consideration (see Fig. 2). The following suggestions for the non-published strains are supported by the calculated values: a) strains JC637, JC742, JC819 and HD01 belong to the species *S. lacunae*, b) TBK1r belongs to a separate novel species, c) SV_7m_r belongs to a separate novel species, c) K23_9 belongs to a separate novel species (the strain was lost and will not be effectively described), d) JC731 belongs to a separate novel species, e) JC639^T^ (the here described strain) belongs to a separate species. The provisional names “*Stieleria magnilauensis*”, “*Stieleria bergensis*” and “*Stieleria marina*” have been mentioned for TBK1r, SV_7m_r and K23_9, respectively, but have not been effectively published yet (or cannot be published due to loss of growth in case of strain K23_9) (Wiegand et al. 2020). Strain JC731 has not been mentioned in any publication so far.

### Pangenome reconstruction and comparison of *N*-acyl amino acid biosynthetic gene clusters

**Fig. 5:**
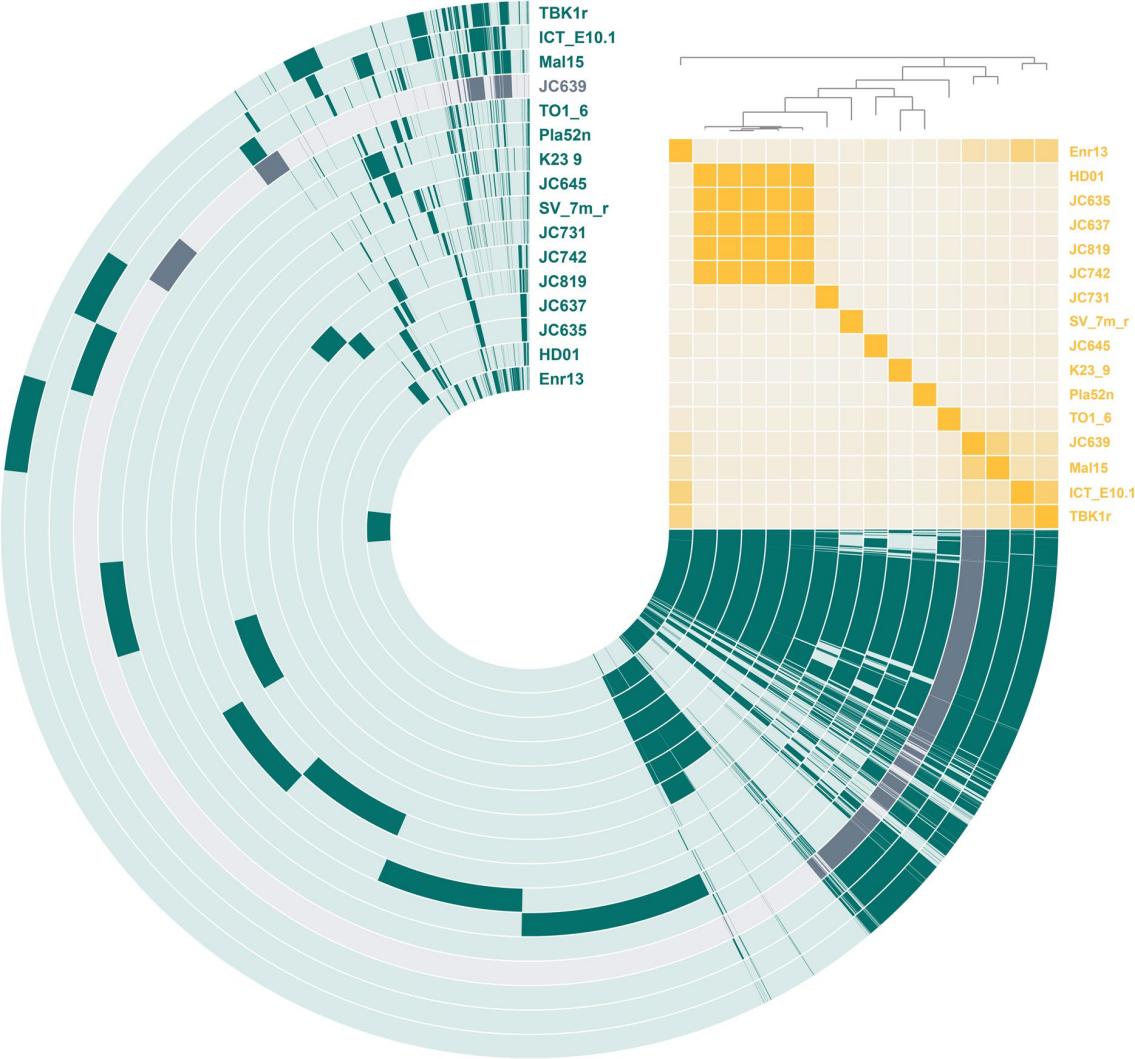
Pangenome based on the analysed genomes. Each open circle represents the pangenome of all strains but is colored darker when the gene is present in the respective genome. The matrix in the upper right corner indicates the degree of relationship based on average nucleotide identity values: ≤ 70% (pale orange) to 100% (bright orange)

In addition to the conclusions on the degree of relationship based on phylogenetic markers, a pangenome of all 16 genomes was constructed to investigate the numbers of conserved core genes and genes with accessory functions. The pangenome based on the available genomes of members of the genera *Stieleria*/*Roseiconus* yielded a total of 28,832 gene clusters (Fig. 5). Among these, 1,676 genes can be found in all analysed genomes, of which 89% (1,485 genes) are associated with a putative function based on the automated gene annotation (Table S8). Many of the identified genes are involved in essential functions, including catabolism, protein biosynthesis, DNA replication, translation, etc., while the list also includes genes coding for proteins with regulatory functions, transporter activities and specialized metabolic traits, e.g. a bacterial microcompartment involved in the catabolism of rhamnose and fucose (Erbilgin et al. 2014). The phylogenetic tree in the pangenome based on ANI values substantiates the results obtained during comparison of the phylogenetic markers. The degree of relationship is also reflected in conserved genes in subgroups, e.g. when comparing the ANI matrix results and shared genome content of strains JC635, JC637, JC742, JC819 and HD01. The analysis of singleton genes exclusive for the here described isolate JC639^T^ yielded 671 hits, of which 143 have a putative annotation (Table S9). Amongst other postulated functions, the hits include putative enzymes of a CRISPR/Cas system, alternative sigma factors and DNA-modifying enzymes.

Two members of the genus *Stieleria*, *S. maiorica* Mal15^T^ and *S. neptunia* Enr13^T^ have been shown to produce *N*-acylated tyrosine derivatives named stieleriacines (Kallscheuer et al. 2020b; Sandargo et al. 2020). The proposed hallmark protein for their biosynthesis is an *N*-acyl amino acid synthase (NasY family enzyme) that ligates the amino acid to the activated fatty acid. To check for potential additional stieleriacine producers, the analysed genomes were inspected for biosynthetic gene clusters that harbour putative *nasY* genes. The genome analysis using antiSMASH yielded 21 BGCs potentially associated with *N*-acyl amino acid biosynthesis. With three *nasY*-containing clusters, *S. neptunia* Enr13^T^ showed the highest number, while strain K23_9, *S. varia* Pla52n^T^ and *S. tagensis* TO1_6^T^ lack such clusters. When using the reported *nasY* cluster of *S. maiorica* Mal15 as reference (Kallscheuer et al. 2020b) (first row in Fig. S3), related clusters were found in *S. neptunia* Enr13^T^, *S. nitratireducens* JC645^T^ and the here presented isolate JC639^T^, identifying these as additional stieleriacine producers. Other clusters with conserved NasY-encoding genes are probably involved in the synthesis of other yet undiscovered *N*-acyl amino acids (Fig. S3).

## Conclusion

Strain JC639^T^ shows clear distinction from its closest relatives *S. maiorica* Mal15^T^ and *S. neptunia* Enr13^T^ based on phylogenetic markers (Fig. 2) and phenotypic features (Table 1). Together, the data justifies the introduction of a novel species of the genus *Stieleria*. The overall phylogenomic comparison of all members of the genera *Stieleria* and *Roseiconus* supports the treatment of all members as a single genus. Hence, we propose to include the two *Roseiconus* species in the genus *Stieleria*.

### Description of *Stieleria mannarensis *sp. nov.

*mannarensis* (man.na.ren’sis. N.L. fem. adj. *mannarensis*, referring to the Gulf of Mannar from which the type strain was isolated).

Aerobic chemoheterotroph with pale pink-pigmented cells. Cells are pear-shaped to ovoid, form aggregates or grow as single cells. The average cell size is 1.5 ± 0.2 × 1.1 ± 0.2 μm (length x width). Cells divide asymmetrically (by budding). NaCl is required for growth and concentrations of up to 7% (w/v) are tolerated. Optimum pH and temperature for growth are 8.0 (range 7.0–10.0) and 25 °C (range 10–30 °C), respectively. D-glucose, lactose, rhamnose, mannose, sucrose, pyruvate, galactose, maltose, and D-xylose were used as carbon and energy source. Fructose, starch, mannitol, malic acid, ascorbate, inositol, sodium acetate, propionate, fumarate, succinate and sorbitol did not support growth. Ammonium sulphate, yeast extract, sodium nitrate, L-leucine, L-tyrosine, L-arginine, L-glutamic acid, L-serine, L-proline, L-methionine, DL-alanine, and cysteine were used as nitrogen sources for growth. Peptone, glycine, L-phenylalanine, L-lysine, L-isoleucine, L-tryptophan, L-aspartic acid, L-glutamine, DL-threonine, DL-ornithine, and urea did not support growth when used as nitrogen source. The API ZYM kit analysis gave positive activity results for alkaline phosphatase, esterase (C4), esterase lipase (C8), leucine arylamidase, valine arylamidase, acid phosphatase, naphthol-AS-BI-phosphohydrolase, β-galactosidase, and α-glucosidase and no activity for lipase (C14), cysteine arylamidase, trypsin, α-chymotrypsin, α-galactosidase, β-glucuronidase, β-glucosidase, *N*-acetyl-β-glucosaminidase, α-mannosidase, and α-fucosidase. Major fatty acids are C_16:0,_ C_18:1_ω9c, C_18:0_, C_16:1_ω7c/C_16:1_ω6c and C_15:1_ω8c. Polar lipids include phosphatidylcholine (PC), phosphatidylethanolamine (PE), two unidentified phospholipids (PL1,2) and two unidentified lipids (L1,2). Homospermidine and putrescine are the polyamines formed. The type strain is JC639^T^ (= KCTC 72168^T^ = NBRC 113878^T^). It was isolated from a marine sponge of the genus *Spheciospongia* in the Gulf of Mannar region on India’s southeastern coast. The type strain has a genome size of 9.56 Mbp with a DNA G + C content of 59.5%.

### Description of *Stieleria lacunae* comb. nov.

Basonym: *Roseiconus lacunae* Kumar et al. 2025 . Strain characteristics are as described before (Kumar et al. 2021a). The type strain is JC635^T^ (= KCTC 72164^T^ = NBRC 113875^T^). Additional strains belonging to the species are JC637, JC742, JC819 (unpublished) and HD01 (Gao et al. 2024).

### Description of *Stieleria nitratireducens* comb. nov.

Basonym: *Roseiconus nitratireducens* Kumar et al. 2025. Strain characteristics are as described before (Kumar et al. 2021a). The type strain is JC645^T^ (= KCTC 72174^T^ = NBRC 113879^T^).

### Emended description of the genus* Stieleria*

Characteristics of the genus are as previously described (Kallscheuer et al. 2020a) with the following additions: Cells are pink-pigmented. Genome sizes range from 7 to 11 Mbp. The DNA G + C content ranges from 54 to 60%. Major fatty acids include C_18:1_ω9c and C_16:0_. Major polar lipids include phosphatidylcholine and phosphatidylethanolamine. Major polyamines are spermidine and putrescine.

## Supplementary Information

Below is the link to the electronic supplementary material.

Supplementary file1 (PDF 928 KB)

Supplementary file2 (XLSX 90 KB)

## Data Availability

The GenBank/EMBL/DDBJ accession number for the 16S rRNA gene sequence of strain JC639 is LR132063. This Whole Genome Shotgun project has been deposited at DDBJ/ENA/GenBank under the accession JACEHH000000000. The version described in this paper is version JACEHH010000000.

## Abbreviations

NCBI: National Centre for Biotechnology Information
*g*ANI: Genome average nucleotide identity
AAI: Average amino acid identity
POCP: Percentage of conserved proteins
KCTC: Korean collection for type cultures
NBRC: Biological Resource Centre NITE
