# Supplementary material for: Description of Stieleria mannarensis sp. nov., isolated from a marine sponge, and proposal to include members of the genus Roseiconus in the genus Stieleria
Source: Antonie Van Leeuwenhoek. 2025 Jul 11;118(8):111. doi: 10.1007/s10482-025-02106-8 (PMC12254169; doi:10.1007/s10482-025-02106-8)
Supplement: Supplementary file 1 — Supplementary file1 (PDF 928 KB) [file 10482_2025_2106_MOESM1_ESM.pdf]

Supporting Information

Description of *Stieleria mannarensis* sp. nov., isolated from a marine sponge, and proposal to include members of the genus *Roseiconus* in the genus *Stieleria*

Nicolai Kallscheuer[1,#], Gaurav Kumar[1,2,3,#], Shabbir Ahamad[2], Sandhya Duddeda[2], Chintalapati Sasikala[4], Christian Jogler[1,3*] and Chintalapati Venkata Ramana[2,*]

[1] Department of Microbial Interactions, Institute of Microbiology, Friedrich Schiller University, Jena, 07743, Germany

[2] Department of Plant Sciences, School of Life Sciences, University of Hyderabad, P.O. Central University, Hyderabad 500046, India

[3] Cluster of Excellence Balance of the Microverse, Friedrich Schiller University, Jena, Germany

[4] Bacterial Discovery Laboratory, Centre for Environment, Institute of Science and Technology, JNT University Hyderabad, Kukatpally, Hyderabad-500085, India.

# Authors contributed equally to this work

***** Corresponding authors: Chintalapati Venkata Ramana: cvr449@gmail.com; Christian Jogler: christian.jogler@uni-jena.de

The GenBank/EMBL/DDBJ accession number for the 16S rRNA gene sequence of strain JC639^T is LR132063. The Whole Genome Shotgun project has been deposited at DDBJ/ENA/GenBank under the accession JACEHH000000000. The version described in this paper is version JACEHH010000000.

Abbreviations: NCBI, National Centre for Biotechnology Information; *g*ANI, Genome Average Nucleotide Identity; AAI, Average Amino Acid Identity; POCP, Percentage Of Conserved Proteins; KCTC, Korean Collection for Type Cultures; NBRC, Biological Resource Centre NITE

Carbon and nitrogen source utilization patterns of *Stieleria maiorica* Mal15^T and *Stieleria neptunia* Enr13^T

For *S. maiorica* Mal15^T (ordered from the DSMZ under deposition number DSM 100215^T), starch, maltose, mannose, D-glucose, inositol, sorbitol, lactose, galactose, and D-xylose are good carbon and energy sources that enabled growth. Fumarate, fructose, sodium propionate, mannitol, sucrose, malic acid, benzoic acid, ascorbate, sodium pyruvate, and sodium succinate did not support growth of *S. maiorica* Mal15^T. Nitrogen sources like peptone, sodium nitrate, ammonium sulphate, yeast extract, L-proline, glycine, L-tyrosine, L-lysine, L-methionine, L-phenylalanine, L-serine, DL-threonine, L-glutamine, and L-tryptophan support the growth of the *S. maiorica*, whereas L-isoleucine, L- leucine, L-arginine, and L-glutamic acid did not allow for biomass production when used a nitrogen source. For *S. neptunia* Enr13^T (DSM 100295^T), starch, maltose, sodium propionate, mannose, sucrose, mannitol, D-glucose, sorbitol, galactose, and D-xylose were the tested carbon and energy sources that allowed for growth. Fructose, fumarate, inositol, benzoic acid, malic acid, lactose, sodium pyruvate, ascorbate, and sodium succinate were not used for biomass formation. Peptone, ammonium sulphate, Yeast extract, L-arginine, L-tyrosine, L-proline, glycine, L-isoleucine, L-lysine, L-methionine, L-phenylalanine, DL-threonine, L-glutamine, and L-tryptophan served as nitrogen sources supporting growth whereas sodium nitrate, L-glutamic acid, L-serine, and L-leucine were not accepted.

Enzymatic activities of strain JC639^T, *S. maiorica* Mal15^T, and *S. neptunia* Enr13^T

Enzymatic activities were determined using the API ZYM kit (BioMérieux). For strain JC639^T, the analysis gave positive results for alkaline phosphatase, esterase (C4), esterase lipase (C8), leucine arylamidase, valine arylamidase, acid phosphatase, naphthol-AS-BI-phosphohydrolase, β-galactosidase, and α-glucosidase while tests were negative for lipase (C14), cysteine arylamidase, trypsin, α-chymotrypsin, α-galactosidase, β-glucuronidase, β-glucosidase, *N*-acetyl-β-glucosaminidase, α-mannosidase, and α-fucosidase. *S. maiorica* Mal15^T was tested positive for alkaline phosphatase, esterase (C4), esterase lipase (C8), leucine arylamidase, valine arylamidase, cysteine arylamidase, trypsin, acid phosphatase, naphthol-AS-BI-phosphohydrolase, α-glucosidase, β-glucosidase, α-mannosidase, and α-fucosidase amd tested negative for lipase (C14), α-chymotrypsin, α-galactosidase, β-galactosidase, β-glucuronidase, and *N*-acetyl-β-glucosaminidase. *S. neptunia* Enr13^T showed activity for esterase (C4), leucine arylamidase, acid phosphatase, naphthol-AS-BI-phosphohydrolase, and α-mannosidase, but lacked activity for alkaline phosphatase, esterase lipase (C8), lipase (C14), valine arylamidase, cysteine arylamidase, trypsin, α-chymotrypsin, α-galactosidase, β-galactosidase, β-glucuronidase, α-glucosidase, β-glucosidase, *N*-acetyl-β-glucosaminidase, and α-fucosidase.

Supporting Tables

The following supporting Tables are provided in a separate Excel file.

Table S1. Fatty acid composition of JC639^T, *S. maiorica* Mal15^T and *S. neptunia* Enr13^T

Table S2. Sampling locations of strains belonging to the genera *Stieleria* and *Roseiconus*

Table S3. All-vs-all comparisons for percentage of conserved proteins (POCP)

Table S4. All-vs-all comparisons for partial *rpoB* gene similarity

Table S5. All-vs-all comparisons for 16S rRNA gene sequence similarity

Table S6. All-vs-all comparisons for average amino acid identity (AAI)

Table S7. All-vs-all comparisons for average nucleotide identity (ANI)

Table S8. Core genes of all analysed genomes extracted from the pangenome

Table S9. Singleton genes of JC639^T extracted from the pangenome

Supporting Figures

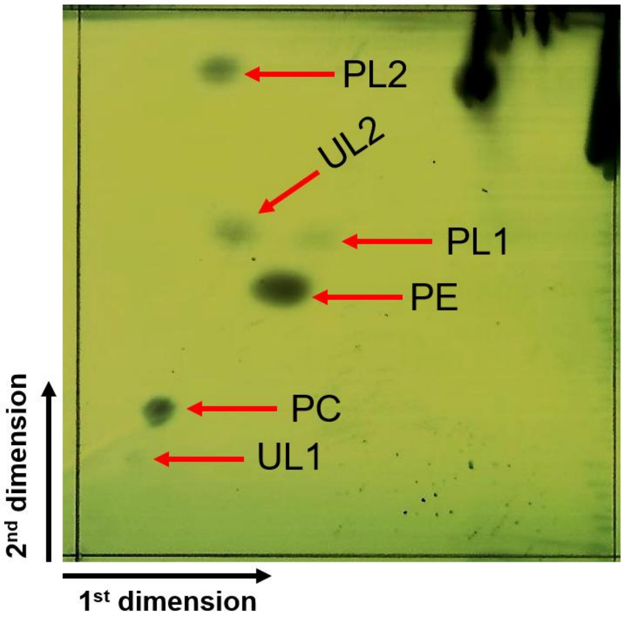

Figure S1. Two dimensional chromatograms showing polar lipids of the strain JC639^T PC, phosphatidylcholine; PL1,2, unidentified phospholipids; PE, phosphatidylethanolamine; UL1,2 unidentified lipids.

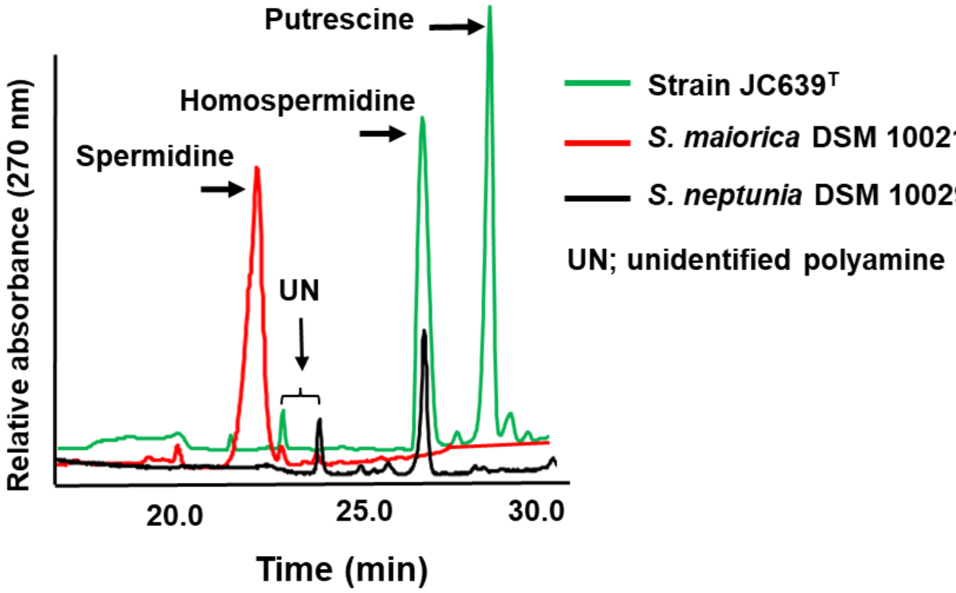

Figure S2. Analysis of polyamines. HPLC chromatogram of polyamines of the strain JC639^T, *S. maiorica* Mal15^T and *S. neptunia* Enr13^T.

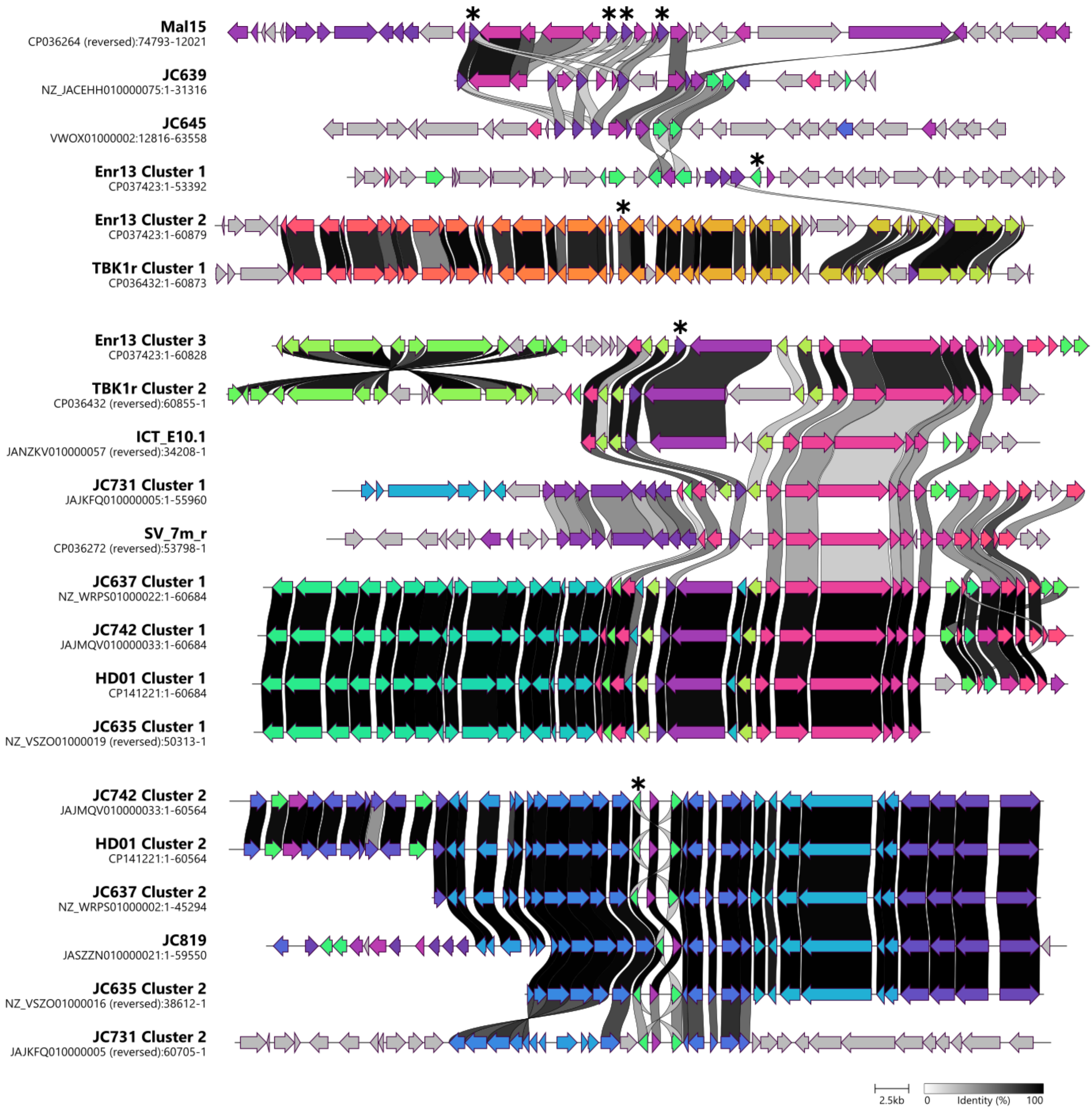

Figure S3. Visualization of *N*-acyl amino acid biosynthetic gene clusters. Biosynthetic gene clusters were predicted with antiSMASH v. 7.1 and visualized with clinker. The reference cluster from *S. maiorica* Mal15^{T} is shown in the first line. Connected genes indicate genes with a similarity above 50% on the protein sequence level. Asterisks indicate putative *N*-acyl amino acid synthase genes.
